# Effect of multistrain probiotics on symptom severity in irritable bowel syndrome: a systematic review and meta-analysis of irritable bowel syndrome–symptom severity score outcomes

**DOI:** 10.1097/MEG.0000000000003074

**Published:** 2025-12-24

**Authors:** Deva Fitra Firdausa Anwar, Zaskia Nafisa Salma, Syavira Dwi Oktaviani, Farhan Naufal Adiyatma, Fadhilah Mahrus Tauhid, Ali Mustofa, Pandit Bagus Tri Saputra, Rumman Karimah, Firas Farisi Alkaff

**Affiliations:** a Faculty of Medicine; b Department of Cardiology and Vascular Medicine, Faculty of Medicine; c Cardiovascular Research and Innovation Center, Universitas Airlangga, Surabaya, East Java, Indonesia; d Faculty of Medicine and Health, Institut Teknologi Sepuluh Nopember, Surabaya, East Java, Indonesia; e Division of Nephrology, Department of Internal Medicine, University Medical Center Groningen, Groningen, The Netherlands; f Division of Pharmacology and Therapy, Department of Anatomy, Histology, and Pharmacology, Faculty of Medicine, Universitas Airlangga, Surabaya, East Java, Indonesia

**Keywords:** irritable bowel syndrome, irritable bowel syndrome–symptom severity score, multistrain, probiotics

## Abstract

Irritable bowel syndrome (IBS) is a common gastrointestinal disorder with varying symptom severity. Although probiotics are frequently used for IBS management, the effects of multistrain probiotic interventions on IBS symptom severity, as measured by the IBS symptom severity score (IBS-SSS), remain unclear. This systematic review, conducted in accordance with the Preferred Reporting Items for Systematic Reviews and Meta-Analyses 2020 guidelines, compared multistrain probiotics with placebo in patients with IBS, focusing on IBS-SSS outcomes. Risk of bias was assessed using the Risk of Bias 2.0 tool, and meta-analysis was performed with Review Manager 5.4.1. From the 1858 screened records, 12 studies involving 1303 participants were included. Multistrain probiotics significantly reduced total IBS-SSS scores [mean difference = −43.66, 95% confidence interval (CI): −65.89 to −21.44, *P* = 0.0001, *I*² = 99%]. Significant improvements were also found in IBS-SSS subscores, including number of days with pain (mean difference = −7.38, 95% CI: −11.86 to −2.89, *P* = 0.001, *I*² = 100%), bloating (−5.62, 95% CI: −10.76 to −0.48, *P* = 0.03, *I*² = 100%), bowel habit satisfaction (−11.90, 95% CI: −19.21 to −4.59, *P* = 0.001, *I*² = 99%), and quality of life (QoL) (−11.99, 95% CI: −16.3 to −7.60, *P* < 0.00001, *I*² = 91%); however, changes in abdominal pain scores and IBS-specific QoL were not statistically significant. High heterogeneity prompted sensitivity analyses to identify contributing factors. Overall, multistrain probiotics significantly reduced IBS-SSS and its subscores but did not significantly improve QoL compared with placebo.

## Introduction

Irritable bowel syndrome (IBS) is a functional gastrointestinal condition marked by recurring abdominal pain, discomfort, and changes in bowel habits. All occurring without the presence of any organic disorder [1]. The Rome Foundation Global Epidemiological Study assessed IBS prevalence in 33 countries, finding similar rates in Europe and the US, with slightly lower rates in Asia and Australia. Egypt showed the highest prevalence. Reported IBS rates were 5.2% (Rome IV) in Gibraltar, 5.9% (Rome III) in the UK, and 6.98% (Rome IV) in Latin America. Data from the US, Canada, and the UK indicated Rome III rates were about double those of Rome IV. IBS prevalence is highest in Africa, similar in the US and Europe, but varies significantly between Europe and Asia. Historically more prevalent in women, IBS is now equally common in Asian men and women. Higher prevalence is noted among the educated, wealthy, students, and younger individuals, with a decline observed with age [2].

Although IBS is highly prevalent, its precise cause remains unclear, and existing treatments typically aim to relieve symptoms [3]. IBS has several types: diarrhea-predominant IBS (IBS-D), constipation-predominant IBS (IBS-C), and mixed IBS (IBS-M). In IBS-D, small intestinal bacterial overgrowth is often present and can be identified through a positive hydrogen breath test. On the other hand, IBS-C is associated with elevated levels of methanogenic archaea, detectable with a positive methane breath test. IBS-M involves fluctuations between diarrhea and constipation [4]. In this article, we will use the IBS symptom severity score (IBS-SSS) as the outcome measurement of the IBS conditions.

The IBS-SSS was created by Francis *et al*. [5] in 1997. This questionnaire is intended to evaluate the perceived severity of IBS symptoms and is commonly used in clinical studies to track the progress of patients with IBS. The IBS-SSS is a composite measure consisting of five questions that cover abdominal pain (including duration and severity), bloating/distension, satisfaction with bowel habits, and IBS-related quality of life (QoL). Each question is scored from 0 to 100, resulting in a total possible score of 500. A score under 75 is typically observed in healthy individuals or those in remission. Scores ranging from 75 to 175 denote mild disease, 175–300 signify moderate disease, and scores above 300 reflect severe disease. It was the pioneering, straightforward approach for tracking disease progression and evaluating treatment efficacy. Over the years, the questionnaire has undergone several iterations and simplifications, although its evolution did not stem from structured patient participation. The IBS-SSS is frequently employed in clinical trials to track disease progression and assess treatment effectiveness [6]. One way to reduce a patient’s IBS-SSS is to use probiotics as an intervention, especially multistrain combinations.

Probiotics are living microorganisms that provide multiple advantages to their host. Their ability to combat bacteria is linked to the production of organic acids, ethanol, hydrogen peroxide, or bacteriocins [7]. Probiotic microorganisms operate through multiple mechanisms, such as enhancing immune function, producing organic acids and antimicrobial substances, interacting with existing gut microbiota, engaging with the host, strengthening the gut barrier, and aiding in enzyme production. Probiotics have shown benefits in treating or preventing various conditions, including diarrhea, IBS, ulcerative colitis, and Crohn’s disease [8]. There are ways of giving probiotic intervention: single-strain probiotic intervention and multistrain probiotic intervention. The number of strains in a probiotic product does not necessarily correlate with increased efficacy. Instead, the choice of probiotics should be based on specific evidence from efficacy trials for particular strains and diseases [9].

The potential influence of multistrain probiotic interventions on the IBS-SSS in patients with IBS has yet to be thoroughly investigated in existing research. Consequently, this review aims to address this gap by investigating the effects of multistrain probiotics on the IBS-SSS in patients with IBS. This study aimed to seek valuable insights and answer the question of how these probiotics influence the severity and progression of IBS as measured by the IBS-SSS.

## Methods

The protocol for this review was registered in the PROSPERO database with the identification number CRD42023452054. This manuscript was prepared according to the Cochrane Collaboration guidelines and adheres to the Preferred Reporting Items for Systematic Reviews and Meta-Analyses Extension (PRISMA) 2020 [10].

### Search approach and data sources

A systematic literature search was conducted across multiple online databases, including Cochrane, PubMed, Sage, Web of Science, Scopus, EbscoHost, ProQuest, Springer, Taylor and Francis, and Wiley, encompassing all publications from inception to June 2024. The literature search involved the utilization of a set of predetermined keywords, which were (Irritable Bowel Syndrome“[Mesh] OR ‘IBS’ OR ‘Irritable Bowel Syndrome Severity Scoring System’ OR ‘IBS-SSS’ OR ‘severity score’ OR ‘scoring’) AND (‘Probiotics’[Mesh] OR ‘multi-strain probiotics’ OR ‘probiotic therapy’ OR ‘probiotic supplements’ OR ‘multi strains’ OR ‘multiple strains’). The keywords employed are outlined in Supplementary Table S1, Supplemental digital content 1, https://links.lww.com/EJGH/B225.

### Criteria for selected studies

The titles and abstracts of the retrieved records were scrutinized for their relevance to our research topic. Subsequently, the full texts of all potentially pertinent articles were meticulously evaluated. All of these procedures were done according to the specified inclusion and exclusion criteria. The inclusion criteria for this study include the following: (a) the population for this study was patients with IBS with any ROME criteria of diagnosis; (b) the intervention was limited to multistrain probiotic therapy; (c) IBS-SSS as an outcome assessed; (d) the study design was restricted to a randomized control trial only. The exclusion criteria include the following: (a) review articles or protocol studies; (b) animal studies; (c) non-English studies; (d) articles’ full text not available.

### Extraction of data

Five independent reviewers (D.F.F.A., Z.N.S., S.D.O., F.N.A., and F.M.T.) conducted meticulous data extraction from each included report to minimize reporting errors. The extracted data were then compared, and any discrepancies were addressed and resolved through discussion until a consensus was reached. Extracted data included: (a) country; (b) study design; (c) population: (d) sample size for both intervention and control; (e) age for both intervention and control; (f) intervention type, administration, and assessment period; (g) control type; (h) intervention duration; (i) result, which extracted all the valuable results in the article. Another table for the primary outcomes (IBS-SSS) details which include: (a) overall IBS-SSS; (b) IBS-SSS QoL; (c) IBS-SSS bloating/abdominal distension; (d) IBS-SSS number of days with pain; (e) IBS-SSS satisfaction/dissatisfaction of bowel habit; (f) IBS-SSS abdominal pain, and also the secondary outcome, which is IBS-QoL, an IBS QoL questionnaire which contains 34 items [11]. More detailed data has been taken by directly contacting the author if needed.

### Included study quality assessment

The quality of the studies included in this analysis was evaluated utilizing specific, appropriate tools. For randomized controlled trials, the Cochrane Risk of Bias 2.0 tool has been employed [12]. The assessment was conducted independently by five reviewers. Any discrepancies that arise among the reviewers have been resolved through discussion. Following this, the assessment results were visualized to provide a clear and comprehensive understanding of the methodological quality of the studies.

### Data synthesis and statistical analysis

Primary quantitative analysis was performed using Review Manager 5.4.1 (Cochrane Collaboration, UK). Outcomes were analyzed with a random effect model because of the presence of heterogeneity [13]. For effect size, the mean difference was used, with a 95% confidence interval (CI) to determine the significance of the overall effect, considering a *P* value of less than 0.05 as statistically significant. For all meta-analysis, forest plots were presented. Heterogeneity was investigated using Higgins’ *I*^2^ value, which indicates heterogeneity can be classified as negligible (0–25%), low (25–50%), moderate (50–75%), or high (>75%) [14]. Any high heterogeneity results have been further analyzed by conducting a sensitivity analysis. If there are uncertain data reporting results, such as the absence of SD (change from baseline value), because such information is often not available in the experiment report and must be inputted again [14]. Missing values were calculated from similar trials that were included in the same meta-analysis. In this case, we first calculated the correlation using the following formula:


$$\begin{array}{c} \text{Corr = }[\left(\text{S}{{\text{D}}^{\text{2}}}_{\text{baseline}}\text{+ S}{{\text{D}}^{\text{2}}}_{\text{follow-up}}\text{‒ S}{{\text{D}}^{\text{2}}}_{\text{change}}\right)\text{/2 }\\ \times \text{ S}{\text{D}}_{\text{baseline}}\;\times \text{ S}{\text{D}}_{\text{follow-up}}]\text{.} \end{array}$$

The SD of the change from the base value is then calculated using the formula [14].


$$\begin{array}{c} \text{S}{{\text{D}}^{\text{2}}}_{\text{change}}\text{= }[\;{\sqrt{\text{S}{\text{D}}^{\text{2}}}}_{\text{baseline}}\text{+ S}{{\text{D}}^{\text{2}}}_{\text{follow-up}}\\ \;\;\;\;\;\;\;\;\;\;\;\;\;\;\;\;\;\;\;\;\;\;\;\;\;\;\;\;\;\;\;\;\;\;\;\;\;\;\;\;\;\;\;\;\text{‒ }\left(\text{2 }\times \text{ Corr }\times \text{ S}{\text{D}}_{\text{baseline}}\;\times \text{ S}{\text{D}}_{\text{follow-up}}\right)\;]\;\text{.} \end{array}$$

The results of the (SD) values that have been obtained are then included along with the mean of each data from each study in the extraction table. The mean and SD change values included in Supplementary Table S2, Supplemental digital content 1, https://links.lww.com/EJGH/B225 are the final results taken from each inclusion study.

## Results

### Study selection and quality assessment

From the mentioned databases, 1858 articles were retrieved. After removing 289 duplicates and screening titles/abstracts, 1569 potential articles were selected. After full-text review, twelve studies consisting of Kajander *et al*. [15]; Williams *et al*. [16]; Simrén *et al*. [17]; Sisson *et al*. [18]; Roberts *et al*. [19]; Ishaque *et al*. [20]; Kim *et al*. [21]; Francavilla *et al*. [22]; Bonfrate *et al*. [23]; Skrzydło-Radomańska *et al*. [24]; Shanshal *et al*. [25]; and Mullish *et al*. [26] were included in the systematic review and meta-analysis. The study selection process is detailed in the PRISMA flow chart (Supplementary Figure S1, Supplemental digital content 1, https://links.lww.com/EJGH/B225). Several studies that seemed to meet the inclusion criteria were excluded due to reasons such as the wrong population, study design, or intervention. A total of 12 studies were quality reviewed using the Cochrane risk-of-bias tool for randomized trials version 2 (RoB 2) as shown in the traffic light plot (Supplementary Figure S2, Supplemental digital content 1, https://links.lww.com/EJGH/B225) and summary (Supplementary Figure S3, Supplemental digital content 1, https://links.lww.com/EJGH/B225).

### Study characteristics

This systematic review and meta-analysis used 12 randomized controlled trial studies as its source of data and information. Published between 2007 and 2024, these studies discuss the effect of multistrain probiotics for patients with IBS focusing on the IBS-SSS as the primary outcome. The complete data extraction and study characteristics can be seen in Supplementary Tables S2 and S3, Supplemental digital content 1, https://links.lww.com/EJGH/B225.

### Effect of multistrain probiotics on total irritable bowel syndrome–symptom severity score

**Fig. 1.**
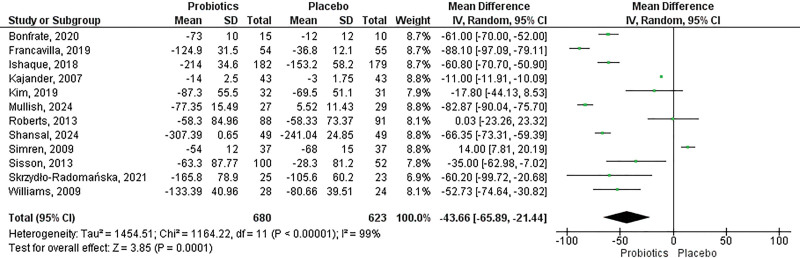
Effect of multistrain probiotics on total IBS-SSS. CI, confidence interval; IBS-SSS, irritable bowel syndrome–symptom severity score.

In the total IBS-SSS parameter, a total of 12 studies comprising 1303 patients compared the effect of multistrain probiotics with placebo in patients with IBS. Overall, there was a significant reduction in IBS-SSS scores with the multistrain probiotics intervention compared with placebo (mean difference = ‐43.66, 95% CI: ‐65.89 to ‐21.44, *P* = 0.0001, *I*^2^ = 99%) (Fig. 1).

### Effect of multistrain probiotics on irritable bowel syndrome–symptom severity score subscores

**Fig. 2.**
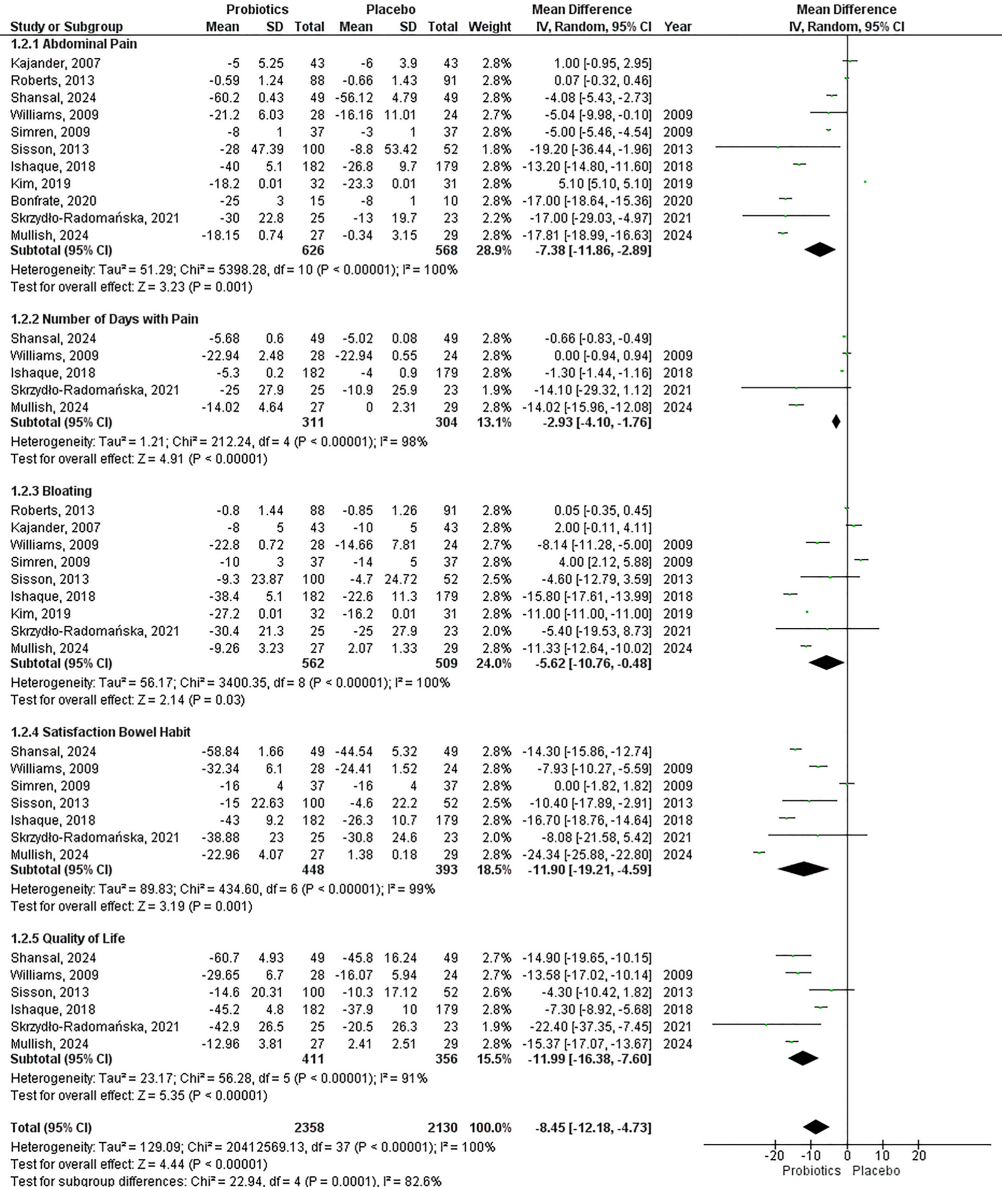
Effect of multistrain probiotics on IBS-SSS subscores. CI, confidence interval; IBS-SSS, irritable bowel syndrome–symptom severity score.

The subscored parameters of the total IBS-SSS score reviewed in this analysis were IBS-SSS abdominal pain, IBS-SSS number of days with pain, IBS-SSS bloating, IBS-SSS satisfaction bowel habit, and IBS-SSS QoL (Fig. 2). There were a total of 12 studies comparing IBS-SSS subscores in multistrain probiotics and placebo interventions, with details of 11 studies for IBS-SSS abdominal pain comprising 1194 patients, five studies for IBS-SSS number of days with pain composed of 615 patients, nine studies for IBS-SSS bloating covering 1071 patients, seven studies for IBS-SSS satisfaction bowel habit consisting of 841 patients, and six studies involving 767 patients for IBS-SSS QoL. Overall, there was a significant decrease in IBS-SSS subscores in the multistrain probiotics intervention compared with placebo (mean difference = ‐8.45, 95% CI: ‐12.18 to ‐4.73, *P* < 0.00001, *I*^2^ = 100%). Subgroup analysis was also performed for each of the subscores. The IBS-SSS abdominal pain score showed a decrease but was NS in the intervention (mean difference = ‐7.38, 95% CI: ‐11.86 to ‐2.89, *P* = 0.001, *I*^2^ = 100%). The IBS-SSS number of days with pain score showed a significant decrease in the multistrain probiotics intervention compared with placebo (mean difference = ‐2.93, 95% CI: ‐4.10 to ‐1.76, *P* < 0.00001, *I*^2^ = 98%). Similarly, other IBS-SSS subscores also significantly decreased in the same intervention comparison, namely in the IBS-SSS bloating subscores, IBS-SSS satisfaction bowel habit, and IBS-SSS QoL (mean difference = ‐5.62, 95% CI: ‐10.76 to ‐0.48, *P* = 0.03, *I*^2^ = 100%; mean difference = ‐11.90, 95% CI: ‐19.21 to ‐4.59, *P* = 0.001, *I*^2^ = 99%; mean difference = ‐11.99, 95% CI: ‐16.38 to ‐7.60, *P* < 0.00001, *I*^2^ = 91%; respectively).

### Effect of multistrain probiotics on irritable bowel syndrome–quality of life

The outcome of QoL was assessed through two separate meta-analyses because of differences in the direction of score interpretation across the included studies. In certain studies, higher scores reflected better QoL, whereas in others, lower scores indicated improvement. Although both scoring approaches captured the same clinical outcome, the inverse numerical interpretation warranted stratification to preserve analytical consistency and interpretative clarity.

**Fig. 3.**
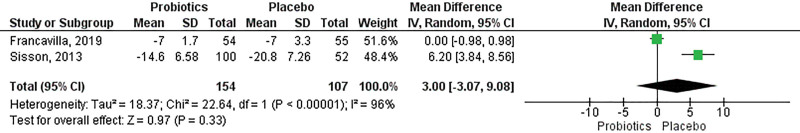
Effect of multistrain probiotics on IBS-QoL in the first subgroup. CI, confidence interval; IBS, irritable bowel syndrome; QoL, quality of life.

**Fig. 4.**
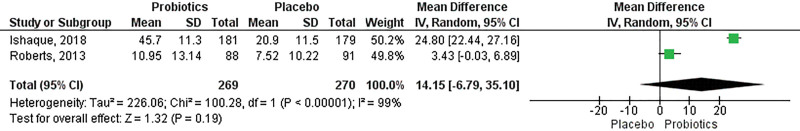
Effect of multistrain probiotics on IBS-QoL in the second subgroup. CI, confidence interval; IBS, irritable bowel syndrome; QoL, quality of life.

In the first subgroup analysis (Fig. 3), two studies comprising a total of 261 participants (154 in the probiotics group and 107 in the placebo group) were included. The pooled analysis demonstrated no statistically significant difference in QoL between the probiotics and placebo groups (mean difference = 3.00; 95% CI: ‐3.07 to 9.08, *P* = 0.33, *I*² = 96%). In the second subgroup (Fig. 4), two studies involving 539 patients assessed QoL using a scale in which higher scores denoted better QoL. The meta-analysis revealed a nonsignificant improvement in QoL among participants receiving multistrain probiotics compared to those receiving placebo (mean difference = 14.15, 95% CI: ‐6.79 to 35.10, *P* = 0.19, *I*^2^ = 99%).

## Discussion

Traditionally, IBS has been regarded as a disorder for which there is no recognized underlying clinical cause for the symptoms that individuals complain of. IBS is characterized by the presence of abnormal bowel patterns together with abdominal pain or discomfort, in the absence of other underlying medical conditions [27]. However, several mechanisms have been proposed as the cause including disruptions in the gut–brain axis, changes in diet, genetic factors, infections and disruptions in the gut microbiota, immune activation, altered intestinal permeability, low-grade mucosal inflammation, abnormalities in serotonin metabolism, and changes in brain function [28].

The evolving understanding of IBS highlights the condition’s multifactorial etiology, which underscores the need to move away from the long-standing misconception of IBS as a purely psychosomatic or functional disorder. The interplay between genetic predispositions, lifestyle factors, and disruptions in the gut–brain axis suggests that IBS is not just localized to the gastrointestinal tract but has systemic implications [29]. Increasing evidence supports the notion that IBS involves bidirectional signaling between the gut microbiota and the central nervous system, referred to as the gut–brain axis, which contributes to its complex symptomatology [30]. Furthermore, disturbances in serotonin metabolism, responsible for modulating gastrointestinal motility and pain perception, represent a key pathophysiological mechanism, especially in light of the higher prevalence of IBS among women [31]. Critically, the emerging role of low-grade mucosal inflammation and altered intestinal permeability introduces an immunological perspective, which could provide novel insights for targeted therapies [32]. These multiple avenues of investigation challenge the reductionist approach of earlier research and emphasize the importance of integrated, multidisciplinary treatment strategies. Although IBS has several classes (IBS-D, IBS-C, and IBS-M), treatment of symptoms should be personalized. Principally, IBS symptoms can be effectively managed by modifying the gut flora through the use of probiotics and antibiotics, avoiding dietary triggers, and modulating the gut–brain axis [33].

Personalized medicine remains the cornerstone for IBS management because of its heterogeneous presentation and multifactorial nature. IBS-D (diarrhea-predominant), IBS-C (constipation-predominant), and IBS-M (mixed type) often respond differently to treatments, reinforcing the necessity for individualized interventions. For example, dietary triggers may play a significant role in IBS-D, whereas laxatives and promotility agents may offer greater benefit in IBS-C [34]. Modifying gut flora through probiotics and antibiotics has garnered significant attention due to its potential to address gut dysbiosis, a prominent feature in IBS [35]. However, it is imperative to critically evaluate these interventions to avoid overtreatment and unintended consequences, such as antibiotic resistance or disruptions to the microbiome. Concurrently, modulating the gut–brain axis through therapies such as cognitive-behavioral therapy (CBT) and hypnotherapy provides a much-needed psychosocial component, emphasizing the mind–gut connection as an essential factor in managing IBS [36]. Combining these approaches reflects the growing consensus that IBS is best addressed through multidisciplinary and evidence-driven treatment strategies.

Single-strain and multistrain probiotics are available as one of the treatments for IBS symptoms. Probiotics are live microorganisms that offer substantial health benefits to the host when given at the appropriate dosage [37], which works by reducing colonization of harmful bacteria and enhancing dysbiosis of the gut microbiota [38]. Ouwehand *et al*. [39] stated that it is generally assumed that more strains will result in a higher likelihood of success with a wider range of efficacy; yet, the current review was unable to locate any solid proof supporting these presumptions. Nonetheless, there is also not any solid proof that the presumptions are false or that a mix of strains exhibits antagonistic actions, as proven by a meta-analysis by [40] which demonstrated that supplementation with multistrain probiotics may be preferable to single-strain formulations in improving IBS symptoms.

The ongoing debate regarding the efficacy of single- versus multistrain probiotics highlights the challenges inherent in probiotic research. Although multistrain probiotics are intuitively assumed to provide broader therapeutic coverage because of their potential synergistic actions, this assumption lacks robust empirical support [39]. The variability in strain composition, dosages, and study designs contributes to conflicting findings, limiting the ability to draw definitive conclusions. Nonetheless, it is plausible that multistrain probiotics offer benefits by mimicking the natural diversity of gut microbiota, which is often disrupted in patients with IBS [41]. By targeting multiple pathways simultaneously – such as enhancing intestinal barrier function, modulating the immune system, and competing with pathogenic bacteria – multistrain formulations may provide a more comprehensive approach [42]. On the other hand, critics argue that combining strains without understanding their interactions risks undermining their efficacy. Moving forward, rigorous trials with standardized formulations are needed to substantiate claims of superiority and clarify whether strain-specific or combined approaches offer the greatest clinical benefit.

### Total irritable bowel syndrome–symptom severity score

Our study included a total of 1303 patients, which showed a significant reduction of 43.66 points in the total IBS-SSS in patients treated with multistrain probiotics compared with placebo. The mechanism behind this might be because of synergy between the various strains in the probiotic formulation since probiotics have a disease- and strain-specific mode of action [41]. Considering the clinically meaningful reduction of greater than or equal to 50 points [5], it is believed that the multistrain probiotics may be a delightful solution for patients with IBS in clinical settings.

The significant reduction in total IBS-SSS scores highlights the clinical potential of multistrain probiotics as a promising intervention for IBS. This observed improvement not only underscores the therapeutic benefit of restoring gut microbial balance but also demonstrates the importance of selecting probiotics with strain-specific efficacy. It is worth noting that the clinical meaningfulness of a greater than or equal to 50-point reduction in IBS-SSS aligns well with patient-centered outcomes, ensuring that the improvement is both statistically and functionally relevant. However, this finding raises questions regarding the sustainability of the benefits and whether long-term administration of probiotics could yield further reductions or prevent symptom recurrence. In addition, while the results are encouraging, the heterogeneity in individual responses must be acknowledged. Future research should aim to identify predictors of response to probiotics, such as baseline gut microbiota composition or genetic markers, to optimize patient selection and maximize treatment outcomes.

### Irritable bowel syndrome–symptom severity score subscores

All symptom subsets of IBS-SSS scores, including abdominal pain severity, significantly decreased, with the greatest decline observed in the number of days with pain. This reduction in IBS-SSS scores aligns with findings from a preceding study that evaluated a human gut-derived multistrain probiotic over 4 weeks and demonstrated improvements across all IBS-SSS domains [43]. The significant decrease across all symptom subscores, including pain severity, frequency, bloating, and bowel habit satisfaction, provides an encouraging indicator of the efficacy of multistrain probiotics in addressing the multifaceted nature of IBS.

From a pathophysiological standpoint, the correlation between IBS-SSS subscores and underlying mechanisms of IBS reflects the interconnected processes at play. The number of days with pain, for instance, correlates strongly with gut motility disturbances and visceral hypersensitivity, which are hallmark features of IBS [44]. Multistrain probiotics may exert their effect by modulating the gut–brain axis, reducing low-grade inflammation, and restoring microbial diversity in the intestinal tract. This improvement in gut microbiota can lead to better regulation of neurotransmitters such as serotonin, which is critical for modulating gastrointestinal motility, pain signaling, and perception of discomfort. Studies have shown that disruptions in serotonin metabolism and receptor activity are linked to increased visceral sensitivity, which contributes to heightened abdominal pain and discomfort in patients with IBS [45]. Therefore, the ability of probiotics to stabilize serotonin levels may help explain their beneficial impact on pain reduction.

The abdominal pain severity subscore, although significant, tends to show variable responses across studies, likely because of differences in probiotic formulations and dosages. Skrzydło-Radomanska’s study highlighted that high doses of 50 billion colony-forming units per day could lead to excessive fermentation of carbohydrates, producing gas and exacerbating symptoms of abdominal discomfort [40]. This underscores the importance of striking a balance between efficacy and tolerability when determining the optimal probiotic dose. Overfermentation and bloating caused by an excessive microbial load can intensify pain, counteracting the intended benefits of probiotics. Mechanistically, abdominal pain is closely tied to the activation of nociceptive pathways in response to mechanical and chemical stimuli within the gut [46]. Probiotics may reduce these nociceptive signals by strengthening the intestinal barrier, preventing the translocation of luminal irritants, and reducing mucosal inflammation – processes that are believed to contribute to visceral hypersensitivity.

Bloating and bowel habit satisfaction subscores, which also significantly improved, reflect additional physiological pathways influenced by probiotics. Bloating is primarily caused by impaired gas handling and altered gut motility, often exacerbated by microbial dysbiosis that leads to excessive fermentation of dietary fibers and undigested carbohydrates [47]. Multistrain probiotics likely address this by rebalancing the gut microbiome, suppressing the overgrowth of gas-producing bacteria, and enhancing the breakdown of fermentable substrates [41]. In addition, probiotics can produce short-chain fatty acids such as butyrate, which contribute to anti-inflammatory effects and promote intestinal homeostasis [48]. By regulating intestinal transit time and improving microbial fermentation efficiency, probiotics may help alleviate bloating and contribute to better bowel movement consistency, which is reflected in improved bowel habit satisfaction scores [49].

The number of days with pain and abdominal discomfort also mirrors the role of immune activation and low-grade inflammation in IBS pathophysiology. Dysbiosis can disrupt the mucosal immune system, leading to increased release of proinflammatory cytokines, such as interleukin 6 (IL-6) and tumor necrosis factor alpha, which sensitize enteric nerves and exacerbate visceral pain [50]. Multistrain probiotics may mitigate this immune activation by promoting the production of anti-inflammatory cytokines like IL-10, thereby reducing gut inflammation and alleviating pain-related symptoms. Furthermore, improved intestinal barrier function achieved through probiotic action prevents the entry of luminal antigens, reducing immune activation and restoring mucosal integrity, ultimately contributing to lower pain frequency and severity [51].

Overall, the significant reduction across all IBS-SSS subscores – including abdominal pain severity, bloating, bowel habit satisfaction, and QoL – highlights the systemic benefits of multistrain probiotics in managing IBS symptoms. These improvements suggest that probiotics can simultaneously address multiple facets of IBS pathophysiology, including dysbiosis, immune dysregulation, altered serotonin metabolism, and visceral hypersensitivity. However, the variability in response across studies indicates the need for personalized probiotic interventions that consider individual microbiota composition, dose optimization, and symptom profiles. Moving forward, a better understanding of the underlying mechanisms through rigorous clinical and mechanistic studies will help clarify the role of probiotics in IBS management and unlock their full therapeutic potential.

By addressing these interconnected pathways, multistrain probiotics provide a comprehensive approach to improving the diverse and often debilitating symptoms of IBS, as captured by the IBS-SSS subscores. This underscores the need for continued research to refine probiotic formulations and elucidate their precise mechanisms of action to deliver consistent, reproducible outcomes for patients with IBS.

### Irritable bowel syndrome–quality of life

QoL in the context of IBS can be assessed through various instruments, each serving distinct purposes and capturing different aspects of the patient experience [52]. The QoL component included as a subscore within the IBS-SSS is a limited, symptom-specific measure that primarily reflects the direct impact of IBS symptoms on daily activities and overall well-being. This subscore provides a more functional and quantitative view of symptom burden, focusing on how symptom severity influences QoL in terms of discomfort, pain, and frequency of disruptions to normal routines [53].

In contrast, the IBS-QoL tool is a dedicated, multidimensional instrument specifically designed to evaluate the broader psychosocial and emotional dimensions of living with IBS. Unlike the IBS-SSS QoL subscore, the IBS-QoL considers a wider range of factors, including the emotional impact of IBS (e.g. anxiety and frustration), social implications (e.g. isolation and embarrassment), and the overall psychological and lifestyle burden of managing a chronic condition [54,55]. This comprehensive approach enables a more nuanced understanding of how IBS affects patients beyond their physical symptoms, capturing the holistic experience of living with the disorder. The difference between these measures lies in their scope and purpose. The IBS-SSS QoL subscore provides a direct reflection of symptom-related disruptions, making it useful for tracking changes in response to treatment. However, it may overlook the complex interplay of psychological, social, and behavioral factors that the IBS-QoL addresses. This distinction may explain why certain studies, including the findings discussed above, show improvements in the QoL subscore of the IBS-SSS but fail to detect significant changes in the broader IBS-QoL measure. These discrepancies underscore the importance of using both symptom-specific and comprehensive QoL tools in clinical trials to capture the full impact of IBS on patients’ lives and evaluate the efficacy of treatments more holistically.

Individuals treated with multistrain probiotics did not exhibit a significant improvement in QoL, although the QoL aspect, which was integrated in the IBS-SSS subscores, showed a statistically significant enhancement. This finding was different to that of earlier studies, demonstrating an improved QoL [56–58]. Despite the fact that generally the multistrain probiotics are assumed to be effective in enhancing QoL, the insignificant result can be explained by the high heterogeneity as well as the highly varied range of values between studies. From all the findings that had already been made, our study remarks that multistrain probiotics may serve as a potential alternative in patients with IBS. The discrepancies observed in QoL outcomes may stem from the subjective nature of QoL assessments and the high variability among patients. Other than that, there are many of the included studies, apart from Francavilla *et al*., Sisson *et al*., Roberts *et al*., and Ishaque *et al*., that do not report IBS-QoL in their findings. This may explain the conflicting results between a significant IBS-SSS subscore reduction and the nonsignificant IBS-QoL results.

QoL in individuals with IBS is shaped by a complex interplay of factors that extend far beyond the severity of physical symptoms [59]. While symptom management is central to improving QoL, factors such as psychological distress, comorbidities, and the ability to maintain social and occupational functioning play equally crucial roles. IBS is closely associated with heightened levels of anxiety, depression, and stress, which often exacerbate symptoms through the gut–brain axis [60]. This bidirectional communication between the central nervous system and the gastrointestinal tract can create a vicious cycle in which psychological distress worsens symptoms, and in turn, debilitating symptoms heighten psychological burdens. Consequently, addressing both the physical and emotional dimensions of IBS is vital for achieving meaningful improvements in QoL.

Probiotics, while effective in alleviating physical symptoms such as abdominal pain, bloating, and altered bowel habits, have shown mixed results in addressing broader psychosocial dimensions of QoL. This may be because of the primarily localized action of probiotics on the gastrointestinal tract, which does not directly address the psychological and social challenges associated with IBS. Although some studies suggest that probiotics can modulate the gut–brain axis by reducing systemic inflammation and producing neuroactive compounds like serotonin and gamma-aminobutyric acid, their impact on psychological well-being and overall QoL remains inconsistent and understudied [61]. For example, while probiotics may help reduce symptom-related distress, they may not be sufficient to mitigate the pervasive anxiety, feelings of social isolation, and disruptions to daily life experienced by patients with IBS [62].

To address these gaps, future clinical trials should adopt a more holistic approach by incorporating validated, IBS-specific QoL assessment tools. Instruments such as the IBS-QoL questionnaire or the Functional Bowel Disorder Severity Index can provide nuanced insights into how IBS impacts various aspects of life, including emotional well-being, energy levels, and the ability to participate in work and social activities [63]. These tools can also help distinguish between improvements in physical symptoms and broader psychosocial outcomes, enabling researchers to better understand the full spectrum of patient needs.

Moreover, exploring the integration of probiotics with psychological interventions could yield synergistic benefits for patients with IBS. CBT, for example, has been shown to be highly effective in managing IBS by helping patients reframe negative thought patterns, develop coping strategies, and reduce stress. Combining CBT with probiotics could address both the physical and psychological components of IBS, creating a more comprehensive treatment approach [64]. Probiotics may help reduce symptom severity and associated distress, while CBT could tackle the emotional and cognitive aspects of the condition, including maladaptive coping mechanisms and fear of symptom flare-ups in social settings [65].

In addition, recognizing and addressing comorbidities, such as fibromyalgia, chronic fatigue syndrome, and migraine, which frequently coexist with IBS, is essential. These comorbid conditions can amplify the overall burden of the disease and further degrade QoL [66]. Probiotics, when used alongside tailored interventions for these comorbidities, may enhance overall patient outcomes. For instance, addressing inflammation and dysbiosis in IBS may also alleviate systemic inflammation contributing to comorbid conditions [67,68].

In conclusion, while probiotics offer promise in mitigating the physical symptoms of IBS, their impact on the broader psychosocial dimensions of QoL remains limited. To achieve more holistic improvements in patient well-being, future studies should prioritize multifaceted interventions that combine probiotics with psychological therapies, lifestyle modifications, and targeted treatments for comorbid conditions. By embracing a multidisciplinary approach, it may be possible to address the full spectrum of challenges faced by patients with IBS and provide them with not just symptom relief but also a significant enhancement in their QoL.

### Limitations and suggestions

This study is the first systematic review and meta-analysis to assess the primary outcomes that are measured objectively, which is IBS-SSS, in patients with IBS that has been given a multistrain probiotic supplement. Therefore, seeking the medication effects of multistrain probiotics in IBS patients with an objective instrument of measure. However, our study is of course not without limitations.

First, the included samples were categorized according to different Rome criteria, from Rome II to Rome IV. This causes the diagnosis in all of the studies included are not made with the same criteria. For example, out of the 12 studies included in the final review, four studies involved patients with ROME II diagnosis, seven studies involved patients with ROME III diagnosis, and one study involved ROME IV diagnosis criteria.

Second, our analysis showed high heterogeneity among studies. Some included studies were also noticed to have a high risk of bias, which must be taken into account in drawing conclusions. As described above, this heterogeneity may result from many factors. These include the diagnostic criteria differences in each of the studies, different ages of the samples involved, different control groups, and various administration of multistrain bacteria types, dosage, and duration of administration. Our analysis revealed high heterogeneity in IBS-QoL measurement in a study where samples exhibited an abnormal level of mental health baseline.

Therefore, in the future, more homogeneous studies are suggested to be conducted to assess the needs for better review results, and an isolated result of a particular multistrain probiotic with a specific dosage and duration of administration can be obtained.

### Conclusion

In conclusion, our systematic review and meta-analysis showed that multistrain probiotics are preferred in terms of improving the overall IBS-SSS and all IBS symptoms significantly. However, no significant differences between both groups in term of IBS-QoL were found. Further studies confirming these results, as well as exploring precise doses and strains effective for different IBS types are needed.

## Acknowledgements

None.

### Conflicts of interest

There are no conflicts of interest.

## Supplementary Material
