# Supplementary material for: Effect of multistrain probiotics on symptom severity in irritable bowel syndrome: a systematic review and meta-analysis of irritable bowel syndrome–symptom severity score outcomes
Source: Eur J Gastroenterol Hepatol. 2025 Dec 24;38(4):397–406. doi: 10.1097/MEG.0000000000003074 (PMC12935185; doi:10.1097/MEG.0000000000003074)
Supplement: Supplementary file 1 [file ejgh-38-397-s001.pdf]

Effect of Multi-Strain Probiotics on Symptom Severity in Irritable Bowel Syndrome: A Systematic Review and Meta-Analysis of IBS-SSS Outcomes

Supplementary Materials

Supplementary Figure 1. PRISMA Flow Chart	Page 2
Supplementary Figure 2. RoB2 Assessment Traffic Light Plot	Page 3
Supplementary Figure 3. RoB2 Assessment Summary	Page 4
Supplementary Table 1. Search strategy	Page 5
Supplementary Table 2. Data Extraction	Page 8
Supplementary Table 3. Study Characteristics	Page 10

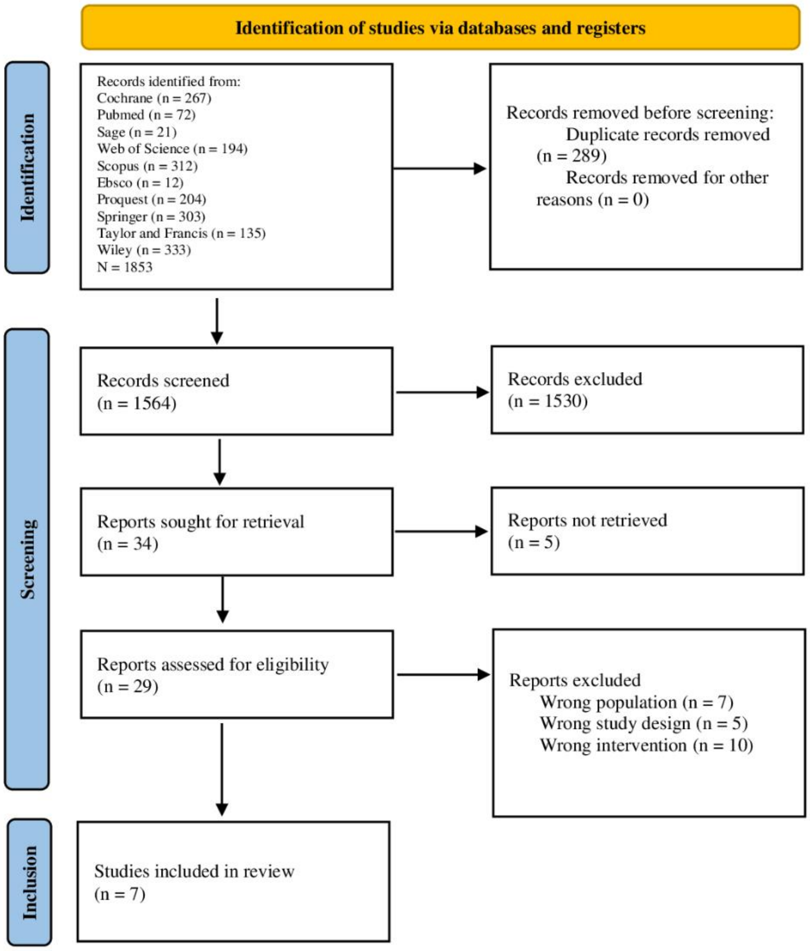

Supplementary Figure 1. PRISMA Flow Chart

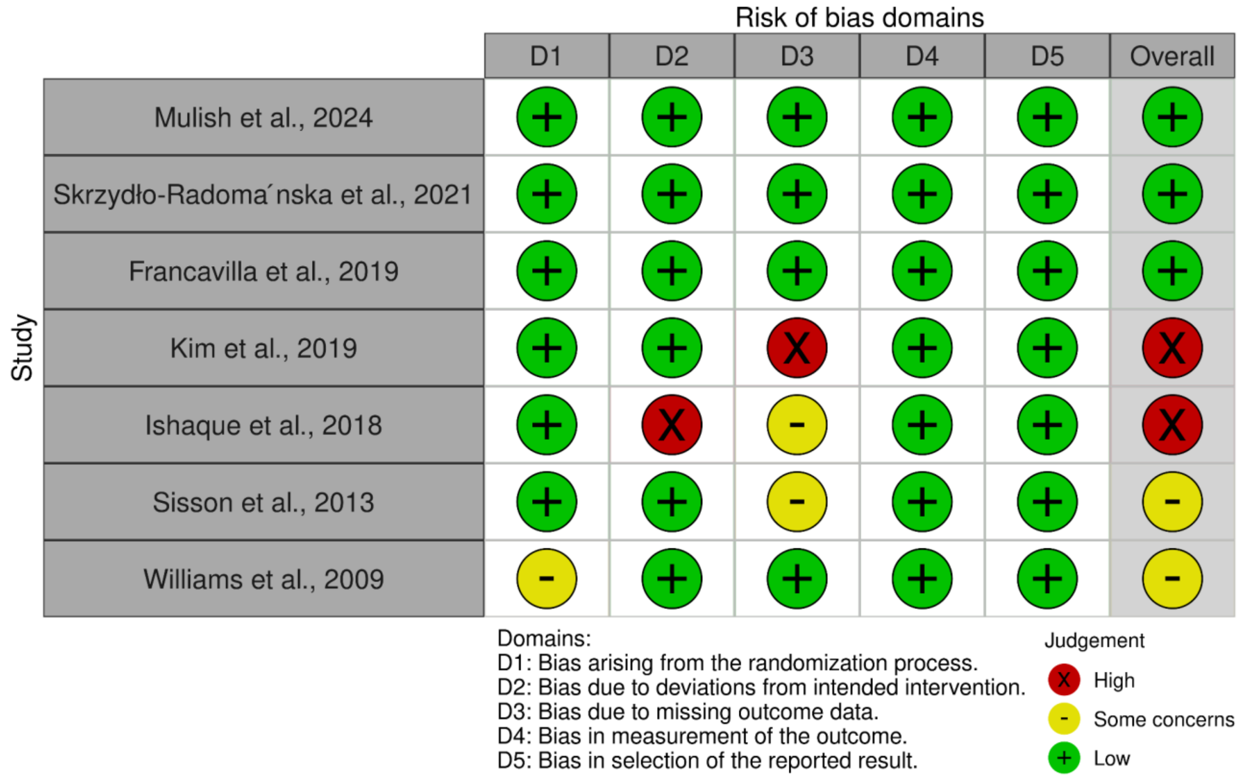

Supplementary Figure 2. RoB2 Assessment Traffic Light Plot

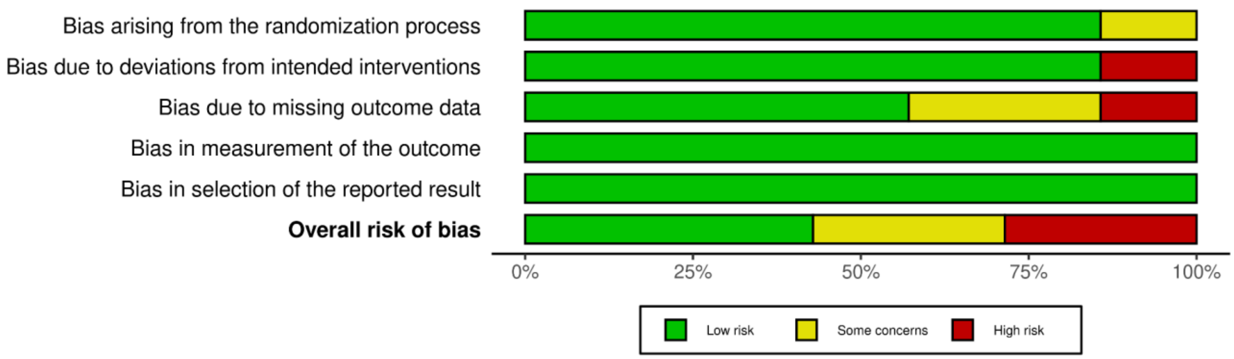

Supplementary Figure 3. RoB2 Assessment Summary

Supplementary Table 1. Search strategy

Database	Keyword
**Cochrane**	(“Irritable bowel syndrome” OR IBS) AND (“Multi strain probiotics” OR probiotics) AND (IBS-SSS OR “ Irritable Bowel Syndrome Severity Scoring System” OR score OR scoring) NOT review<br><br>*limited to English and Trials
**PubMed**	("Irritable Bowel Syndrome"[Mesh] OR IBS) AND (("Probiotics"[Mesh]) OR “multi strain probiotics”) AND (IBS-SSS OR “ Irritable Bowel Syndrome Severity Scoring System” OR score OR scoring) NOT review<br><br>*limited to RCT
**Sage**	(“Irritable bowel syndrome” OR "IBS") AND (“Multi strain probiotics” OR "probiotics") AND ("IBS-SSS" OR “ Irritable Bowel Syndrome Severity Scoring System” OR "score" OR "scoring") NOT review
**Web of Science**	(“Irritable bowel syndrome” OR "IBS") AND (“Multi strain probiotics” OR "probiotics") AND ("IBS-SSS" OR “ Irritable Bowel Syndrome Severity Scoring System” OR "score" OR "scoring") NOT review

**Scopus**	(“Irritable bowel syndrome” OR IBS) AND (“Multi strain probiotics” OR probiotics) AND (IBS-SSS OR “ Irritable Bowel Syndrome Severity Scoring System” OR score OR scoring) NOT review<br><br>*limited to English and research article
**EBSCOhost**	(“Irritable bowel syndrome” OR IBS) AND (“Multi strain probiotics” OR probiotics) AND (IBS-SSS OR “ Irritable Bowel Syndrome Severity Scoring System”) NOT review
**ProQuest**	(“Irritable bowel syndrome” OR IBS) AND (“Multi strain probiotics” OR probiotics) AND (IBS-SSS OR “ Irritable Bowel Syndrome Severity Scoring System” OR score OR scoring) NOT review AND stype.exact("Scholarly Journals")<br><br>*limited to scholarly journals
**Springer**	(“Irritable bowel syndrome” OR IBS) AND (“Multi strain probiotics” OR probiotics) AND (IBS-SSS OR “ Irritable Bowel Syndrome Severity Scoring System” OR score OR scoring) NOT review<br><br>*limited to article

**Taylor n Francis**	(“Irritable bowel syndrome” OR IBS) AND (“Multi strain probiotics” OR probiotics) AND (IBS-SSS OR “ Irritable Bowel Syndrome Severity Scoring System” OR score OR scoring) NOT review<br><br>*limited to article
**JSTOR**	(“Irritable bowel syndrome” OR IBS) AND (“Multi strain probiotics” OR probiotics) AND (IBS-SSS OR “ Irritable Bowel Syndrome Severity Scoring System” OR score OR scoring) NOT review
**Wiley**	(“Irritable bowel syndrome” OR IBS) AND (“Multi strain probiotics” OR probiotics) AND (IBS-SSS OR “ Irritable Bowel Syndrome Severity Scoring System” OR score OR scoring) NOT review<br><br>*limited to journal

Supplementary Table 2. Data Extraction

First Author, Year	Trial Design	Diagnostic criteria	Patients	Age (Years)		Control Type	Intervention Type	Dose	Duration (weeks)	Outcome
				Trial	Control					
Mullish et al., 2024	RCT	ROME IV	*N* = 56, 27 received probiotics	31.30 ± 5.60	32.62 ± 6.34	Placebo (capsules contained microcrystalline cellulose)	Mixture of four strains of probiotics: Lactobacillus acidophilus CUL60 NCIMB 30157; Lactobacillus acidophilus CUL21 (NCIMB 30156); Bifidobacterium bifidum CUL20 (NCIMB 30153); Bifidobacterium animalis subsp. lactis CUL34 (NCIMB 30172); and microcrystalline cellulose.	Total of 25 billion CFU in each capsule. (1 capsule/day)	8	Overall IBS-SSS↓ ($P < 0.0001$)<br>IBS-SSS specific abdominal pain severity↓ ($P < 0.0001$)<br>IBS-SSS specific days with abdominal pain↓ ($P < 0.0001$)<br>IBS-SSS specific bloating severity↓ ($P < 0.0001$)<br>IBS-SSS specific dissatisfaction with bowel habit↓ ($P <$ 0.0001)<br>IBS-SSS specific quality of life↓ ($P < 0.0001$)<br>HADS anxiety score↓ ($P = 0.0002$)<br>HADS depression score↓ ($P < 0.0001$)<br>IBS-BRQ↓ ($P = 0.0002$)<br>BSFS↓ ($P = 0.2909$)<br>Body weight↑ ($P = 0.0111$)<br>BMI↑ ($P = 0.0165$)<br>Systolic blood pressure↑ ($P = 0.2892$)<br>Diastolic blood pressure↓ (P = 0.9542)
Skrzydło-Radomańska et al., 2021	RCT	ROME III	*N* = 48, 25 received probiotics	45.5 ± 11.1	40.7 ± 14.4	Placebo (capsules contained maltodextrin)	Mixture of ten strains of probiotics: four Bifi- dobacterium, five Lactobacillus, and one Streptococcus thermophilus species.	Total of 2.5 billion CFU in each capsule. (2 capsules/day)	8	Overall IBS-SSS↓ ($P = 0.005$)<br>IBS-SSS specific abdominal pain severity↓ ($P = 0.015$),<br>IBS-SSS specific days with abdominal pain↓ ($P = 0.105$)<br>IBS-SSS specific bloating severity↓ ($P = 0.594$)<br>IBS-SSS specific dissatisfaction with bowel habit↓ ($P =$ 0.526)<br>IBS-SSS specific quality of life↓ ($P = 016$)<br>IBS-GIS↑ ($P = 0.003$)
Francavilla et al., 2019	RCT	ROME III	*N* = 109, 54 received probiotics	43.3 ± 10.85	44.6 ± 11.025	Placebo	Mixture of five strains probiotics: Lactobacillus casei LMG 101/37 P-17504, Lactobacillus plantarum CECT 4528, Bifidobacterium animalis subsp. lactis Bi1 LMG P-17502, Bifidobacterium breve Bbr8 LMG P-17501, B. breve Bl10 LMG P-17500	Total of 40 billion CFU in each sachet. (1 sachet/day)	6	Overall IBS-SSS↓ ($P < 0.001$)<br>GSRS↓ ($P < 0.001$)<br>BSFS↓ ($P < 0.01$)<br>IBS-QoL↑ ($P > 0.05$)
Kim et al., 2019	RCT	ROME II	*N* = 63, 32 received probiotics	18-75 years old	18-75 years old	Placebo (capsule contained maltodextrin)	Mixture of five strain of probiotics: Bifidobacterium longum BORI, Bifidobacterium bifidum BGN4, Bifidobacterium lactis AD011, Bifidobac- terium infantis IBS007, and Lactobacillus acidophilus AD031.	Total of 5 billion viable cells in a lyophilized powder form with the other ingredients including maltodextrin, corn starch, and silicon dioxide in each capsule. (3 capsules/day)	8	Overall IBS-SSS↓ ($P > 0.05$)<br>IBS-SSS specific abdominal pain severity↓ ($P > 0.05$)<br>IBS-SSS specific days with abdominal pain↓ ($P > 0.05$)<br>IBS-SSS specific bloating severity↓ ($P > 0.05$)<br>IBS-SSS specific dissatisfaction with bowel habit↓ ($P =$ 0.423)<br>IBS-SSS specific quality of life↓ ($P = 0.431$)<br>Urinary levels of PAME and PEA↑ ($P < 0.001$)<br>IBS-QoL↑ ($P = 0.011$)
Ishaque et al., 2018	RCT	ROME III	*N* = 360, 181 received probiotics	32.2 ± 10.1	31.7 ± 9.7	Placebo (capsules contained microcrystalline	Mixture of 14 strains of probiotics: Bacillus subtilis PXN 21, Bifidobacterium spp. (B. bifidum PXN 23, B. breve PXN 25, B. infantis.	Total of 2 billion CFU per capsule. (2 capsules/twice daily)	16	Overall IBS-SSS↓ ($P < 0.001$)<br>IBS-SSS specific abdominal pain severity↓ ($P < 0.001$)<br>IBS-SSS specific days with abdominal pain↓ ($P < 0.001$)<br>IBS-SSS specific bloating severity↓ ($P < 0.001$)

						cellulose in a vegetable capsule made of hydroxypropyl methylcellulose)	PXN 27, B. longum PXN 30), Lactobacillus spp. (L.acidophilus PXN 35, L. delbrueckii spp. Bulgaricus PXN39, L. casei PXN 37, L. plantarum PXN 47, L. rhamnosus PXN 54, L.helveticus PXN 45, L. salivarius PXN 57), Lactococcus lactis PXN 63, and Streptococcus thermophilus PXN 66.			IBS-SSS specific dissatisfaction with bowel habit↓ (P < 0.001)<br>IBS-SSS specific quality of life↓ (P = 0.015)<br>Number of bowel motions↓ (P < 0.001)<br>Passing excess mucus↓ (P = 0.272)<br>IBS-QoL↓ (P < 0.001)
Sisson et al., 2013	RCT	ROME III	N = 152, 100 received probiotics	39.1 ± 10.5	36.8 ± 10.8	Placebo	Mixture of four strains of probiotics: Lactobacillus rhamnosus NCIMB 30174; Lactobacillus plantarum NCIMB 30173; Lactobacillus acidophilus NCIMB 30175; Enterococcus faecium NCIMB 30176.	Total of 10 billion live bacteria in each 50 ml dose residing in a of water-based barley extract suspension. (1 ml/kg/day)	12	Overall IBS-SSS↓ (P = 0.01)<br>IBS-SSS specific abdominal pain severity↓ (P = 0.03)<br>IBS-SSS specific bloating severity↓ (P = 0.23)<br>IBS-SSS specific dissatisfaction with bowel habit↓ (P = 0.01)<br>IBS-SSS specific quality of life↓ (P = 0.16)<br>IBS-QoL↑ (P = 0.47)<br>Urinary lactulose↓ (P > 0.9)<br>Faecal calprotein↓ (P = 0.57)
Williams et al., 2009	RCT	ROME II	N = 52, 28 received probiotics	40 ± 12	38 ± 11	Placebo (capsule identically packaged with cellulose. 300mg Maltodextrin)	Mixture of four strains of probiotics; L. acidophilus, CUL-60 (NCIMB 30157), CUL-21(NCIMB 30156), B. bifidum CUL-20 (NCIMB 30153) and B. lactis CUL-34 (NCIMB 30172).	Total of 25 billion CFU in each capsule. (1 capsule/day)	8	Overall IBS-SSS↓ (P = 0.0217)<br>IBS-SSS specific abdominal pain severity↓ (P = 0,0028)<br>IBS-SSS specific days with abdominal pain↓ (P < 0.05)<br>IBS-SSS specific bloating severity↓ (P < 0.05)<br>IBS-SSS specific dissatisfaction with bowel habit↓ (P < 0.05)<br>IBS-SSS specific quality of life↓ (P < 0.05)

BMI, Body Mass Index; BSFS, Bristol Stool Form Score; CFU, Colony Forming Unit; GSRS, Gastrointestinal Symptom Rating Scale; HADS, Hospital Anxiety and Depression Scale; IBS, Irritable Bowel Syndrome; IBS-BRQ, Irritable Bowel Syndrome Behavioral Responses Questionnaire; IBS-GIS, Irritable Bowel Syndrome Global Improvement Scale; IBS-SSS, Irritable Bowel Syndrome Severity Scoring System; IBS-QoL, Irritable Bowel Syndrome Quality of Life; PAME, Palmitic Acid Methyl Ester; PEA, Palmitoleoyl Ethanolamide; RCT, Randomized Controlled Trial.

Supplementary Table 3. Study Characteristics

First Author, Year				Williams et al., 2009	Sisson et al., 2013	Ishaque et al., 2018	Kim et al., 2019	Francavilla et al., 2019	Skrzydło-Radomańska et al., 2021	Mullish et al., 2024
Outcome	Total IBS-SSS		Pre-Intervention	283 ± 60.59	303.6 ± 67.75	333.0 ± 40.4	207.9 ± 132.1	295 ± 84.9	339.6 ± 76.8	249.68 ± 59.00
			Post-Intervention	150.23 ± 101.96	240.2 ± 109.18	115.2 ± 75.0	120.6 ± 76.6	170.1 ± 53.4	173.8 ± 60.9	172.33 ± 74.49
			Different	-133.39 ± 40.96	-63.3 ± 87.77	-214 ± 34.6	87.3 ± 55.5	-124.9 ± 31.5	-165.8 ± 78.9	-77.35 ± 15.49
			Pre-Control	252 ± 59.92	306 ± 80.31	332.9 ± 42.0	204.4 ± 150.3	237.6 ± 86.5	325.9 ± 50.7	275.17 ± 53.57
			Post-Control	172.00 ± 99.51	272 ± 102.17	179.7 ± 100.2	134.9 ± 99.2	200.8 ± 74.4	220.3 ± 55.7	280.69 ± 65.01
			Different	-80.66 ± 39.51	-28.3 ± 81.20	-153.2 ± 58.2	-69.5 ± 51.1	-36.8 ± 12.1	-105.6 ± 60.2	5.52 ± 11.43
	Subgroup of IBS-SSS	Abdominal Pain	Pre-Intervention	44.50 ± 18.03	106.3 ± 40.63	58.5 ± 11.1	42.5 ± 11.4	N/A	63.0 ± 21.8	60.37 ± 14.54
			Post-Intervention	27.31 ± 21.09	81.9 ± 56.46	18.1 ± 15.2	19.2 ± 11.4	N/A	33.0 ± 13.9	42.22 ± 15.28
			Different	-21.2 ± 6.03	-28.0 ± 47.39	-40.4 ± 4.1	-18.2 ± 0.01	N/A	−30.0 ± 22.8	-18.15 ± 0.74
			Pre-Control	32.79 ± 15.04	111.7 ± 42.31	57.2 ± 10.6	35.5 ± 8.9	N/A	55.4 ± 19.9	65.17 ± 14.30
			Post-Control	24.73 ± 23.59	100.8 ± 53.78	30.2 ± 19.9	17.3 ± 8.9	N/A	42.4 ± 17.6	64.83 ± 17.45
			Different	-16.16 ± 11.01	-8.8 ± 53.42	-27.0 ± 9.3	-23.3 ± 0.01	N/A	−13.0 ± 19.7	-0.34 ± 3.15
		Number of Days with Pain	Pre-Intervention	48.64 ± 21.81	N/A	7.7 ± 2.3	N/A	N/A	43.0 ± 22.3	30.42 ± 13.23
			Post-Intervention	28.27 ± 21.09	N/A	2.2 ± 1.9	N/A	N/A	18.0 ± 19.8	16.40 ± 8.59
			Different	-22.94 ± 2.48	N/A	-5.5 ± 0.4	N/A	N/A	−25.0 ± 27.9	-14.02 ± 4.64
			Pre-Control	42.67 ± 23.74	N/A	8.1 ± 2.3	N/A	N/A	33.7 ± 20.8	29.31 ± 10.69
			Post-Control	32.14 ± 22.52	N/A	3.9 ± 3.0	N/A	N/A	22.8 ± 21.2	29.31 ± 13.00
			Different	-22.94 ± 0.55	N/A	-4.2 ± 0.7	N/A	N/A	−10.9 ± 25.9	0.00 ± 2.31
		Bloating	Pre-Intervention	48.54 ± 25.77	73.8 ± 18.52	58.5 ± 11.5	37.4 ± 15.1	N/A	58.0 ± 31.2	62.96 ± 13.25
			Post-Intervention	36.65 ± 23.51	44.4 ± 29.17	19.6 ± 15.8	21.2 ± 15.1	N/A	29.0 ± 20.0	40.00 ± 17.32
			Different	-22.8 ± 0.72	-9.3 ± 23.87	-38.9 ± 4.3	-27.2 ± 0.01	N/A	−30.4 ± 21.3	-9.26 ± 3.23
			Pre-Control	46.71 ± 21.83	68.4 ± 21.23	58.9 ± 12.0	40.2 ± 18.9	N/A	66.3 ± 26.8	63.79 ± 15.45
			Post-Control	39.27 ± 25.00	48.2 ± 28.27	35.9 ± 23.5	13.0 ± 18.9	N/A	35.9 ± 19.7	65.17 ± 15.26
			Different	-14.66 ± 7.81	-4.7 ± 24.72	-23.0 ± 11.5	-16.2 ± 0.01	N/A	−25.0 ± 27.9	2.07 ± 1.33
		Satisfaction Bowel Habit	Pre-Intervention	73.39 ± 17.73	53.4 ± 24.39	71.0 ± 9.7	N/A	N/A	86.4 ±17.0	44.44 ± 28.19
			Post-Intervention	48.38 ± 20.01	58.5 ± 24.12	26.5 ± 19.1	N/A	N/A	47.5 ± 19.2	35.19 ± 31.42
			Different	-32.34 ± 6.1	-15.0 ± 22.63	-44.5 ± 9.4	N/A	N/A	−38,88 ± 23.0	-22.96 ± 4.07
			Pre-Control	68.04 ± 20.08	54.5 ± 25.61	69.6 ± 13.1	N/A	N/A	88.2 ± 16.5	57.59 ± 20.29
			Post-Control	48.68 ± 17.15	63.2 ± 18.44	42.2 ± 25.0	N/A	N/A	57.4 ± 14.8	59.66 ± 21.63
			Different	-24.41 ± 1.52	-4.6 ± 22.20	-17.4 ± 11.9	N/A	N/A	−30.8 ± 24.6	1.38 ± 0.18
		Quality of Life	Pre-Intervention	67.61 ± 15.70	70.2 ± 17.38	68.6 ± 12.6	N/A	N/A	89.2 ± 18.8	51.48 ± 17.25
			Post-Intervention	48.58 ± 19.81	55.4 ± 23.47	23.4 ± 17.4	N/A	N/A	46.3 ± 21.5	38.52 ± 21.07
			Different	-29.65 ± 6.7	-14.6 ± 20.31	-45.2 ± 4.8	N/A	N/A	−42.9 ± 26.5	-12.96 ± 3.81
			Pre-Control	61.88 ± 11.64	70.7 ± 19.90	66.1 ± 11.1	N/A	N/A	82.3 ± 20.0	59.31 ± 16.02
			Post-Control	48.59 ± 14.40	59.8 ± 20.63	28.2 ± 20.1	N/A	N/A	61.8 ± 18.3	61.72 ± 18.53
			Different	-16.07 ± 5.94	-10.3 ± 17.12	-37.9 ± 10	N/A	N/A	20.5 ± 26.3	2.41 ± 2.51
	IBS-QoL		Pre-Intervention	N/A	53.2 ± 20.25	22.6 ± 10.5	N/A	33.7 ± 17	N/A	N/A
			Post-Intervention	N/A	53.4 ± 20.52	68.3 ± 21.8	N/A	26.7 ± 18.7	N/A	N/A
			Different	N/A	6.9 ± 13.21	45.7 ± 11.3	N/A	-7 ± 1.7	N/A	N/A
			Pre-Control	N/A	50.7 ± 19.93	27.5 ± 13.0	N/A	31.5 ± 19.3	N/A	N/A
			Post-Control	N/A	51.2 ± 20.24	48.4 ± 24.5	N/A	24.5 ± 16	N/A	N/A
			Different	N/A	5.3 ± 13.63	20.9 ± 11.5	N/A	-7 ± 3.3	N/A	N/A

N/A, Not Available; IBS-SSS, Irritable Bowel Syndrome Severity Scoring System; IBS-QoL, Irritable Bowel Syndrome Quality of Life
